# Supplementary material for: Towards efficient glaucoma screening with modular convolution-involution cascade architecture
Source: PeerJ Comput Sci. 2025 Apr 21;11:e2844. doi: 10.7717/peerj-cs.2844 (PMC12192679; doi:10.7717/peerj-cs.2844)
Supplement: Supplemental Information 3 [file peerj-cs-11-2844-s003.docx]

| Algorithm 1 Pseudo code of the hybrid MCICNet-LightGBM model | |
| --- | --- |
| **Require:** Retinal fundus image dataset $D={\{(X_{i},y_{i})\}}_{i=1}^{N}$, where $X_{i}$ represents the input image and $y_{i}$ the corresponding label | |
| **Ensure:** Trained MCICNet-LightGBM model | |
| 1: | **Feature Extraction with MCICNet** |
| 2: | Initialize MCICNet with input $X\in R^{96\times96\times3}$ |
| 3: | Apply Residual Multi-scale Feature Fusion Module: $X\leftarrow RMFFM(X)$ |
| 4: | **for** each $k$ in {32, 64, 128, 256} **do** |
| 5: | Configure involution parameters: group number, kernel size, stride, reduction ratio |
| 6: | Apply Residual Hybrid Convolutional-Involutional Module: $X\leftarrow RHCIM(X, k)$ |
| 7: | **end for** |
| 8: | Extract feature tensor $F=Flatten(X)$ |
| 9: | **Train LightGBM Classifier** |
| 10: | Prepare training set: $F_{train}=extract\_features(MCICNet, X_{train})$ |
| 11: | Prepare validation set: $F_{val}=extract\_features(MCICNet, X_{val})$ |
| 12: | Prepare test set: $F_{test}=extract\_features(MCICNet, X_{test})$ |
| 13: | Convert to LightGBM dataset: |
| 14: | $D_{train}=\{(F_{i},y_{i})\vert i\in train set\}$ |
| 15: | $D_{val}=\{(F_{i},y_{i})\vert i\in val set\}$ |
| 16: | Train LightGBM model: |
| 17: | $LGBM\leftarrow lgb.train(D_{train},D_{val})$ |
| 18: | **Evaluate Model Performance** |
| 19: | Predict on test set |
| 20: | Compute metrics |
| 21: | **return** Trained MCICNet-LightGBM model |
