# Supplementary material for: Towards efficient glaucoma screening with modular convolution-involution cascade architecture
Source: PeerJ Comput Sci. 2025 Apr 21;11:e2844. doi: 10.7717/peerj-cs.2844 (PMC12192679; doi:10.7717/peerj-cs.2844)
Supplement: Supplemental Information 4 [file peerj-cs-11-2844-s004.docx]

Table S1 Model performance metrics for different seeds on the test set of LAG dataset.

| **Model** | **Seed** | **Precision** | **Sensitivity** | **Specificity** | **F1-score** | **Accuracy** | **MCC** | **AUC** |
| --- | --- | --- | --- | --- | --- | --- | --- | --- |
| SqueezeNet | Seed A | 0.8622 | 0.8935 | 0.8220 | 0.8675 | 0.8724 | 0.7549 | 0.9731 |
|  | Seed B | 0.8933 | 0.9030 | 0.8949 | 0.8976 | 0.9053 | 0.7962 | 0.9696 |
|  | Avg | 0.8778 | 0.8983 | 0.8584 | 0.8826 | 0.8889 | 0.7756 | 0.9714 |
| AlexNet | Seed A | 0.8644 | 0.8837 | 0.8686 | 0.8715 | 0.8792 | 0.7477 | 0.9467 |
|  | Seed B | 0.8565 | 0.8800 | 0.9105 | 0.8639 | 0.8710 | 0.7361 | 0.9482 |
|  | Avg | 0.8605 | 0.8819 | 0.8896 | 0.8677 | 0.8751 | 0.7419 | 0.9475 |
| MobileNetV2 | Seed A | 0.8953 | 0.8902 | 0.9322 | 0.8927 | 0.9026 | 0.7872 | 0.9712 |
|  | Seed B | 0.8966 | 0.8885 | 0.8405 | 0.8923 | 0.9026 | 0.7850 | 0.9696 |
|  | Avg | 0.8959 | 0.8894 | 0.8864 | 0.8925 | 0.9026 | 0.7861 | 0.9704 |
| DenseNet121 | Seed A | 0.9057 | 0.9187 | 0.9153 | 0.9114 | 0.9176 | 0.8244 | 0.9684 |
|  | Seed B | 0.9086 | 0.8713 | 0.7743 | 0.8855 | 0.8998 | 0.7790 | 0.9706 |
|  | Avg | 0.9072 | 0.8950 | 0.8448 | 0.8985 | 0.9087 | 0.8017 | 0.9695 |
| ResNet18 | Seed A | 0.8966 | 0.8959 | 0.9280 | 0.8962 | 0.9053 | 0.7923 | 0.9683 |
|  | Seed B | 0.9041 | 0.8692 | 0.7743 | 0.8826 | 0.8971 | 0.7725 | 0.9659 |
|  | Avg | 0.9004 | 0.8826 | 0.8512 | 0.8894 | 0.9012 | 0.7824 | 0.9671 |
| GoogLeNet | Seed A | 0.8898 | 0.8833 | 0.9300 | 0.8864 | 0.8971 | 0.7680 | 0.9578 |
|  | Seed B | 0.8904 | 0.8824 | 0.8327 | 0.8862 | 0.8971 | 0.7728 | 0.9619 |
|  | Avg | 0.8901 | 0.8829 | 0.8814 | 0.8863 | 0.8971 | 0.7704 | 0.9599 |
| ShuffleNet | Seed A | 0.9063 | 0.9077 | 0.9322 | 0.9070 | 0.9149 | 0.8140 | 0.9699 |
|  | Seed B | 0.9171 | 0.9178 | 0.8949 | 0.9174 | 0.9245 | 0.8349 | 0.9708 |
|  | Avg | 0.9117 | 0.9128 | 0.9136 | 0.9122 | 0.9197 | 0.8244 | 0.9704 |
| EfficientNetB0 | Seed A | 0.9104 | 0.9090 | 0.9386 | 0.9097 | 0.9176 | 0.8100 | 0.9671 |
|  | Seed B | 0.8971 | 0.8527 | 0.7393 | 0.8687 | 0.8861 | 0.7485 | 0.9590 |
|  | Avg | 0.9038 | 0.8809 | 0.8389 | 0.8892 | 0.9019 | 0.7792 | 0.9631 |
| VGG16 | Seed A | 0.9291 | 0.9298 | 0.9492 | 0.9294 | 0.9355 | 0.8592 | 0.9793 |
|  | Seed B | 0.8563 | 0.8792 | 0.9066 | 0.8637 | 0.8710 | 0.7351 | 0.9471 |
|  | Avg | 0.8927 | 0.9045 | 0.9279 | 0.8966 | 0.9033 | 0.7972 | 0.9632 |
| ViT | Seed A | 0.9262 | 0.9231 | 0.8949 | 0.9246 | 0.9314 | 0.8493 | 0.9790 |
|  | Seed B | 0.9222 | 0.9293 | 0.9222 | 0.9255 | 0.9314 | 0.8515 | 0.9797 |
|  | Avg | 0.9242 | 0.9262 | 0.9086 | 0.9251 | 0.9314 | 0.8504 | 0.9793 |
| MaxViT | Seed A | 0.9169 | 0.9075 | 0.8638 | 0.9119 | 0.9204 | 0.8244 | 0.9707 |
|  | Seed B | 0.9051 | 0.8984 | 0.8560 | 0.9015 | 0.9108 | 0.8034 | 0.9719 |
|  | Avg | 0.9110 | 0.9029 | 0.8599 | 0.9067 | 0.9156 | 0.8139 | 0.9713 |
| SwinT | Seed A | 0.9297 | 0.9289 | 0.9066 | 0.9293 | 0.9355 | 0.8587 | 0.9808 |
|  | Seed B | 0.9394 | 0.9371 | 0.9144 | 0.9382 | 0.9437 | 0.8765 | 0.9818 |
|  | Avg | 0.9345 | 0.9330 | 0.9105 | 0.9337 | 0.9396 | 0.8676 | 0.9813 |
| MCICNet-NoInvolution | Seed A | 0.9493 | 0.9452 | 0.9682 | 0.9472 | 0.9519 | 0.8934 | 0.9887 |
|  | Seed B | 0.9404 | 0.9427 | 0.9300 | 0.9416 | 0.9465 | 0.8832 | 0.9895 |
|  | Avg | 0.9448 | 0.9439 | 0.9491 | 0.9444 | 0.9492 | 0.8883 | 0.9891 |
| **Proposed MCICNet** | Seed A | 0.9515 | 0.9491 | 0.9682 | 0.9503 | 0.9547 | 0.9000 | 0.9914 |
|  | Seed B | 0.9545 | 0.9521 | 0.9339 | 0.9533 | 0.9574 | 0.9066 | 0.9912 |
|  | Avg | 0.9530 | 0.9506 | 0.9511 | 0.9518 | 0.9561 | 0.9033 | 0.9913 |
