# Supplementary material for: Towards efficient glaucoma screening with modular convolution-involution cascade architecture
Source: PeerJ Comput Sci. 2025 Apr 21;11:e2844. doi: 10.7717/peerj-cs.2844 (PMC12192679; doi:10.7717/peerj-cs.2844)
Supplement: Supplemental Information 5 [file peerj-cs-11-2844-s005.docx]

Table S2 Model performance metrics for different seeds on the test set of ACRIMA dataset.

| **Model** | **Seed** | **Precision** | **Sensitivity** | **Specificity** | **F1-score** | **Accuracy** | **MCC** | **AUC** |
| --- | --- | --- | --- | --- | --- | --- | --- | --- |
| SqueezeNet | Seed A | 0.8599 | 0.8560 | 0.7333 | 0.8411 | 0.8411 | 0.7159 | 0.9691 |
|  | Seed B | 0.9150 | 0.8982 | 0.9667 | 0.9035 | 0.9065 | 0.8131 | 0.9840 |
|  | Avg | 0.8874 | 0.8771 | 0.8500 | 0.8723 | 0.8738 | 0.7645 | 0.9765 |
| AlexNet | Seed A | 0.8879 | 0.8686 | 0.9500 | 0.8741 | 0.8785 | 0.7562 | 0.9447 |
|  | Seed B | 0.9072 | 0.9028 | 0.9047 | 0.9333 | 0.9065 | 0.8100 | 0.9663 |
|  | Avg | 0.8976 | 0.8857 | 0.9274 | 0.9037 | 0.8925 | 0.7831 | 0.9555 |
| MobileNetV2 | Seed A | 0.9348 | 0.9172 | 0.9833 | 0.9228 | 0.9252 | 0.8518 | 0.9904 |
|  | Seed B | 0.9455 | 0.9408 | 0.9667 | 0.9428 | 0.9439 | 0.8863 | 0.9872 |
|  | Avg | 0.9402 | 0.9290 | 0.9750 | 0.9328 | 0.9346 | 0.8691 | 0.9888 |
| DenseNet121 | Seed A | 0.9072 | 0.9028 | 0.9333 | 0.9047 | 0.9065 | 0.8100 | 0.9759 |
|  | Seed B | 0.9137 | 0.9181 | 0.9000 | 0.9151 | 0.9158 | 0.8318 | 0.9840 |
|  | Avg | 0.9105 | 0.9105 | 0.9167 | 0.9099 | 0.9112 | 0.8209 | 0.9800 |
| ResNet18 | Seed A | 0.8880 | 0.8839 | 0.9167 | 0.8856 | 0.8878 | 0.7718 | 0.9624 |
|  | Seed B | 0.9046 | 0.9098 | 0.8833 | 0.9059 | 0.9065 | 0.8143 | 0.9599 |
|  | Avg | 0.8963 | 0.8969 | 0.9000 | 0.8958 | 0.8972 | 0.7931 | 0.9612 |
| GoogLeNet | Seed A | 0.8290 | 0.8316 | 0.8333 | 0.8300 | 0.8317 | 0.6605 | 0.8863 |
|  | Seed B | 0.7766 | 0.7654 | 0.8500 | 0.7684 | 0.7757 | 0.5419 | 0.8521 |
|  | Avg | 0.8028 | 0.7985 | 0.8417 | 0.7992 | 0.8037 | 0.6012 | 0.8692 |
| ShuffleNet | Seed A | 0.9455 | 0.9408 | 0.9667 | 0.9428 | 0.9439 | 0.8863 | 0.9801 |
|  | Seed B | 0.9183 | 0.9112 | 0.9500 | 0.9140 | 0.9158 | 0.8294 | 0.9631 |
|  | Avg | 0.9319 | 0.9260 | 0.9583 | 0.9284 | 0.9299 | 0.8579 | 0.9716 |
| EfficientNetB0 | Seed A | 0.9569 | 0.9491 | 0.9833 | 0.9522 | 0.9532 | 0.9060 | 0.9890 |
|  | Seed B | 0.9537 | 0.9514 | 0.9667 | 0.9525 | 0.9532 | 0.9051 | 0.9720 |
|  | Avg | 0.9553 | 0.9503 | 0.9750 | 0.9524 | 0.9532 | 0.9056 | 0.9805 |
| VGG16 | Seed A | 0.8574 | 0.8612 | 0.8500 | 0.8586 | 0.8598 | 0.7185 | 0.9674 |
|  | Seed B | 0.8951 | 0.8968 | 0.9000 | 0.8959 | 0.8971 | 0.7919 | 0.9695 |
|  | Avg | 0.8763 | 0.8790 | 0.8750 | 0.8773 | 0.8785 | 0.7552 | 0.9685 |
| ViT | Seed A | 0.9493 | 0.9385 | 0.9833 | 0.9425 | 0.9439 | 0.8877 | 0.9908 |
|  | Seed B | 0.9550 | 0.9533 | 0.9333 | 0.9534 | 0.9532 | 0.9072 | 0.9933 |
|  | Avg | 0.9521 | 0.9459 | 0.9583 | 0.9479 | 0.9486 | 0.8974 | 0.9921 |
| MaxViT | Seed A | 0.9043 | 0.9074 | 0.9000 | 0.9055 | 0.9065 | 0.8117 | 0.9773 |
|  | Seed B | 0.9493 | 0.9385 | 0.9833 | 0.9425 | 0.9439 | 0.8877 | 0.9819 |
|  | Avg | 0.9268 | 0.9229 | 0.9417 | 0.9240 | 0.9252 | 0.8497 | 0.9796 |
| SwinT | Seed A | 0.9346 | 0.9324 | 0.9500 | 0.9334 | 0.9345 | 0.8670 | 0.9752 |
|  | Seed B | 0.9329 | 0.9348 | 0.9333 | 0.9337 | 0.9345 | 0.8677 | 0.9922 |
|  | Avg | 0.9337 | 0.9336 | 0.9417 | 0.9335 | 0.9345 | 0.8674 | 0.9837 |
| MCICNet-NoInvolution | Seed A | 0.9421 | 0.9477 | 0.9167 | 0.9435 | 0.9439 | 0.8897 | 0.9940 |
|  | Seed B | 0.9455 | 0.9408 | 0.9667 | 0.9428 | 0.9439 | 0.8863 | 0.9826 |
|  | Avg | 0.9438 | 0.9442 | 0.9417 | 0.9431 | 0.9439 | 0.8880 | 0.9883 |
| **Proposed MCICNet** | Seed A | 0.9647 | 0.9598 | 0.9833 | 0.9619 | 0.9626 | 0.9244 | 0.9943 |
|  | Seed B | 0.9621 | 0.9621 | 0.9667 | 0.9621 | 0.9626 | 0.9241 | 0.9972 |
|  | Avg | 0.9634 | 0.9609 | 0.9750 | 0.9620 | 0.9626 | 0.9242 | 0.9957 |
